# Obesity‐induced skeletal muscle remodeling: A comparative analysis of exercise training and ACE‐inhibitory drug in male mice

**DOI:** 10.14814/phy2.16025

**Published:** 2024-04-29

**Authors:** Ana Beatriz Proença, Beatriz Alexandre‐Santos, Isabele Gomes Giori, Jaime Silva Filho Alex‐Marques, Clarice Machado‐Santos, Marcus Machado, D'Angelo Carlo Magliano, Antonio Claudio Lucas da Nobrega, Eliete Dalla Corte Frantz

**Affiliations:** ^1^ Laboratory of Exercise Sciences, Biomedical Institute Fluminense Federal University Niteroi Rio de Janeiro Brazil; ^2^ Research Center on Morphology and Metabolism, Biomedical Institute Fluminense Federal University Niteroi Rio de Janeiro Brazil; ^3^ Laboratory of Teaching and Research in Histology and Compared Embryology Fluminense Federal University Niteroi Rio de Janeiro Brazil; ^4^ Biomedical Science Department Ross University School of Veterinary Medicine Basseterre St. Kitts & Nevis; ^5^ National Institute for Science & Technology—INCT Physical (in)Activity & Exercise, CNPq Niteroi Rio de Janeiro Brazil

**Keywords:** enalapril, exercise training, obesity, renin–angiotensin system, skeletal muscle

## Abstract

Obesity over‐activates the classical arm of the renin‐angiotensin system (RAS), impairing skeletal muscle remodeling. We aimed to compare the effect of exercise training and enalapril, an angiotensin‐converting enzyme inhibitor, on RAS modulation in the skeletal muscle of obese animals. Thus, we divided C57BL/6 mice into two groups: standard chow (SC) and high‐fat (HF) diet for 16 weeks. At the eighth week, the HF‐fed animals were divided into four subgroups—sedentary (HF), treated with enalapril (HF‐E), exercise training protocol (HF‐T), and combined interventions (HF‐ET). After 8 weeks of treatment, we evaluated body mass and index (BMI), body composition, exercise capacity, muscle morphology, and skeletal muscle molecular markers. All interventions resulted in lower BMI and attenuation of overactivation in the classical arm, while favoring the B2R in the bradykinin receptors profile. This was associated with reduced apoptosis markers in obese skeletal muscles. The HF‐T group showed an increase in muscle mass and expression of biosynthesis markers and a reduction in expression of degradation markers and muscle fiber atrophy due to obesity. These findings suggest that the combination intervention did not have a synergistic effect against obesity‐induced muscle remodeling. Additionally, the use of enalapril impaired muscle's physiological adaptations to exercise training.

## Graphical abstract

Enalapril and aerobic exercise training have been found to shift the activity of the renin‐angiotensin system (RAS) toward the counterregulatory arm and the kallikrein‐kinin system (KKS) toward the bradykinin receptor subtype 2 (B2R), which in turn helps reduce apoptosis markers (BAX/Bcl‐2 ratio) and improve PGC‐1α expression in muscle. However, it is worth noting that only exercise training mitigates obesity‐induced myofiber atrophy. This is due to its ability to upregulate the mTOR/p70S6K pathway, boost muscle mitochondrial biogenesis marker (PGC‐1α), and lower levels of muscle protein degradation markers (MuRF‐1 and Atrogin‐1). It is important to note that AT1R (angiotensin type 1 receptor), MasR (Mas receptor), Bax (Bcl‐2‐associated X protein), Bcl‐2 (B‐cell lymphoma 2), and UPP (ubiquitin‐proteasome pathway) are all relevant abbreviations in this context; also, green arrows represent activate regulation, and red arrows represent the inhibitory regulation.

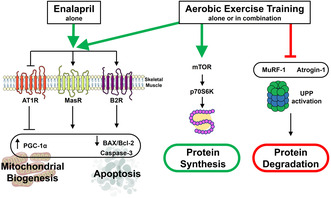

## 1 INTRODUCTION

The renin‐angiotensin system (RAS) is a major hormonal system that regulates blood pressure, fluid balance, and electrolyte balance in the body. It consists of two distinct arms: the classical and the counterregulatory (Powers et al., 2018; Sepúlveda‐Fragoso et al., 2021). In the classic arm, renin and angiotensin‐converting enzyme (ACE) cleave angiotensin to produce angiotensin I (Ang I) and angiotensin II (Ang II), respectively. Ang II primarily exerts its effects through the Ang type 1 receptor (AT1R), leading to vasoconstriction, fibrosis, and insulin resistance (Basso & Terragno, 2001; Santos et al., 2018). On the other hand, in the counterregulatory arm, ACE2 cleaves Ang II into Ang (Alexandre‐Santos et al., 2022; Basso & Terragno, 2001; Frantz et al., 2018; Nilsson et al., 2013; Powers et al., 2018; Santos et al., 2018; Sepúlveda‐Fragoso et al., 2021), which then acts on the Mas receptor (MasR). MasR has vasodilatory and anti‐inflammatory functions and can improve insulin sensitivity in skeletal muscle (Alexandre‐Santos et al., 2022; Frantz et al., 2018; Santos et al., 2018).

Several studies have indicated that muscle atrophy due to obesity is triggered by the upregulation of the classical RAS pathway (Frantz et al., 2018; Nilsson et al., 2013). Both Ang II and ACE are believed to contribute to skeletal muscle atrophy by impairing protein biosynthesis and mitochondrial biogenesis. Moreover, they over‐activate myonuclear apoptosis and the ubiquitin‐proteasome pathway (UPP), which is one of the primary proteolytic systems involved in the degradation of sarcomeric proteins (Cabello‐Verrugio et al., 2017; Frantz et al., 2018; Lóry et al., 2019). The activation of RAS through AT1R has been shown to raise the level of various pro‐inflammatory mediators in the plasma, which in turn promotes skeletal muscle atrophy (Cabello‐Verrugio et al., 2017; Frantz et al., 2017; Powers et al., 2018). On the other hand, treatment with Ang (Alexandre‐Santos et al., 2022; Basso & Terragno, 2001; Frantz et al., 2018; Nilsson et al., 2013; Powers et al., 2018; Santos et al., 2018; Sepúlveda‐Fragoso et al., 2021) has been found to counteract muscle atrophy through the insulin‐like growth factor (IGF)‐1/protein kinase B (Akt). Akt activates the mammalian protein target rapamycin (mTOR), which is critical in controlling muscle biosynthesis through the activation of p70 ribosomal protein S6 kinase (p70S6K) (Cabello‐Verrugio et al., 2017; Morales et al., 2016). Apart from its anti‐atrophic effects, the Ang (Alexandre‐Santos et al., 2022; Basso & Terragno, 2001; Frantz et al., 2018; Nilsson et al., 2013; Powers et al., 2018; Santos et al., 2018; Sepúlveda‐Fragoso et al., 2021)/MasR arm has antiapoptotic effects, and it also reduces the expression of muscle atrophy F‐box gene (Atrogin‐1/MAFbx) and muscle RING‐68 finger protein (MuRF‐1) induced by Ang II in myotubes (Cabello‐Verrugio et al., 2017; Cisternas et al., 2015).

The modulation of RAS in skeletal muscle has been studied as a potential target for preventing skeletal muscle remodeling caused by various diseases. Studies have shown that ACE inhibitors (ACEi) can improve endothelial and mitochondrial function, which can significantly reduce the production of muscle reactive oxygen species (ROS) (Kakutani et al., 2021; Lin et al., 2022). These findings suggest that ACEi may exert their effects, at least in part, through the kallikrein‐kinin system (KKS) by increasing the action of bradykinin on B2 kinin receptors (B2R), which play a role in the regulation of skeletal muscle. While B2R is expressed in the muscular system, B1R expression promotes obesity and is linked to muscle degradation in myocytes through Atrogin‐1 and MuRF‐1‐dependent mechanisms (Henriksen & Jacob, 2003; Reis et al., 2015). Studies have shown that enalapril, an ACEi drug, primarily acts peripherally, affecting the RAS in tissues outside the central nervous system. By inhibiting ACE, enalapril reduces the production of Ang II locally in these tissues, which may enhance muscle quality and potentially preserve muscle function by attenuating apoptosis (Carter et al., 2011; Santos et al., 2008).

Aerobic exercise training is a highly recommended nonpharmacological approach to improve the health and body composition of individuals who have obesity and skeletal muscle disorders (Beals et al., 2019; Heo et al., 2018; Machado et al., 2017). Previous studies have shown that aerobic exercise training can modulate the RAS, favoring its counterregulatory arm (Frantz et al., 2017; Magalhães et al., 2020; Motta‐Santos et al., 2016), reducing several inflammatory markers, improving insulin resistance, and promoting mitochondrial biogenesis and skeletal muscle hypertrophy (Frantz et al., 2018; Giori et al., 2021; Heo et al., 2018). However, the impact of enalapril, exercise, and their associated interventions on skeletal muscle remodeling in cases of obesity are still unclear. To address this, the current study aimed to evaluate the effect of three interventions in animals fed a high‐fat (HF) diet: (1) treatment with enalapril (HF‐E), (2) treatment with aerobic exercise training (HF‐T), and (3) treatment with a combination of the enalapril and aerobic training treatments (HF‐ET). The study aimed to examine the morphological and molecular mechanisms of obesity‐induced muscle remodeling. The hypothesis was that ACEi and exercise training would reduce similarly the obesity‐induced skeletal muscle remodeling.

## 2 MATERIALS AND METHODS

### 2.1 Animals and diet

All the experimental procedures adhered to the standard guidelines for animal experimentation (National Institute of Health Guide, 8th edition, 2011) and were approved by the Animal Ethics Committee of the Fluminense Federal University (Protocol number CEUA 2504060718). Male C57BL/6 mice had free access to food and water while being housed in a controlled environment with a temperature of 23 ± 2°C, relative humidity of 60 ± 5%, and a 12/12 h light/dark cycle.

At the age of 12 weeks, the animals were randomly divided into two groups: SC (*n* = 10, 10% of energy from lipids) and HF diet (*n* = 40, 50% of energy from lipids) for 8 weeks. All diets were manufactured with purified nutrients by PragSoluções (Jaú, São Paulo, Brazil), following recommendations of the American Institute of Nutrition (Reeves et al., 1993), which are detailed in Table S1. After 8 weeks of controlled diet, the animals that were fed a high‐fat diet were randomly divided into four groups (*n* = 10 per group): sedentary (HF), treated with enalapril (HF‐E), submitted to an aerobic exercise training (HF‐T), and combined interventions with enalapril and exercise training (HF‐ET). The animals in the SC group did not undergo any interventions. The body mass index (BMI) was calculated using the formula: BM/nose‐to‐anus length (NAL).

### 2.2 Enalapril treatment

During 8 weeks, an HF diet was followed to establish an obesity model. After this period enalapril treatment began and continued for another 8 weeks. Enalapril was mixed into the manufactured diet at a dose of 10 mg/kg/day (E6888; Sigma‐Aldrich, MO), which is widely used in the literature (Machackova et al., 2004; Santos et al., 2008).

### 2.3 Assessment of maximal aerobic capacity and exercise training protocol

All animals, including sedentary animals, were first adapted to a motor‐driven rodent treadmill (Imbramed®; RS, Brazil) at low velocity. The adaptation involved running at a speed of 0.2 km/h, 0% slope, 10 min/day, for three consecutive days.

After the adaptation period was over, all the groups underwent a Maximal Exercise Running Test (MERT), to determine their individual maximal exercise capacity. This test allowed the researchers to adjust the intensity of exercise training. The test was initiated at 0.2 km/h, followed by increments of 0.1 km/h every 3 min, with a 0% slope. The test ended when the animals got exhausted and could no longer run, remaining at the end of the mat on the shock grid for more than 5 s. The shock grid had bars that delivered low electrical currents of approximately 2 mA, which caused minor discomfort but no harm to the animals. The MERT was conducted during pretraining (8th week), middle (12th week, to adjust the intensity—data not shown), and post‐training (16th week). The time to exhaustion was measured, and the data were presented at pretraining, post‐training, and as the difference (delta) between them.

After 8 weeks of a HF diet, the animals underwent an 8‐week aerobic exercise training protocol. They were trained on a motor‐driven rodent treadmill, for 60 min each day, 5 days a week at a moderate intensity equivalent to 60% of the maximal velocity obtained during the MERT, as previously described (Giori et al., 2021).

### 2.4 Body composition

At Week 16, a dual‐energy X‐ray absorptiometry (DEXA) (Lunar IDXA 200368; GE Health Care, WI) was used to determine the animals' body fat and lean mass percentage. The Encore 2008 software (version 12.20; GE Health Care) was used to quantify to calculate the body composition.

The animals were anesthetized (ketamine, 40 mg/kg IP; xylazine, 5 mg/kg IP) and positioned in ventral decubitus under the scanning area. The equipment scanned the animals along a sagittal line that passed under the center of specific anatomical points, such as the skull, spine, and pelvis.

### 2.5 Tissue extraction

Seventy‐two hours after the last training session, the mice were deprived of food for 6 h and then deeply anesthetized with intraperitoneal ketamine (80 mg/kg) and xylazine (10 mg/kg). The quadriceps femoris and triceps surae (gastrocnemius and soleus) muscles were dissected. Five of the total mice in each group were used for protein expression analysis. Their muscle tissue was rapidly frozen and stored at −80°C freezer. The tissue of the remaining five mice was embedded into freshly prepared fixative (formaldehyde 4% w/v, 0.1 M phosphate buffer pH 7.2) for further histological analysis.

### 2.6 Muscle tissue stereology

The quadriceps muscle samples were fixed in formalin and then embedded in paraffin. After that 5‐μm‐thick slices were stained with hematoxylin and eosin. Digital images of the slides were then scanned and quantified using ScanScopeTM CS (Aperio Technologies, CA). To measure the myofiber cross‐sectional area, at least ten random microscopic fields were analyzed from each of the five animals per group. About 50 myofibers were measured in each animal to ensure complete visualization of cytoplasmic membranes.

For stereological analysis, the volume density (VV [myofibers]) and numerical density (Qa [myofibers]) of myofiber were estimated by counting points using the STEPanizer Web‐based software. The relative volume occupied by myofibers in the entire skeletal muscle (including the contractile and noncontractile area in percentage) was used to estimate myofiber volume: Vv [myofibers] = PP [myofibers]/PT. Where PP represents the number of points that hit the myofiber, while PT represents the total number of test points in a test system comprising 36 test points. The numerical density was estimated by counting the myofiber number in a frame of known area (mm^2^) from 10 slides per animal (*n* = 5 animals/group) (Mandarim‐de‐Lacerda et al., 2010).

### 2.7 Immunohistochemistry

Samples of the quadriceps femoris muscle (*n* = 5) were fixed and embedded in paraffin. 5‐μm‐thick sections were then subjected to antigen retrieval by treating with citrate buffer pH 6.0 for 45 min at 96°C. The sections were then blocked using peroxidase and protein block solution (RE7140‐CE, Novolink Polymer Detection Systems Kit; Leica Biosystems, RU). Primary antibodies (1:200) for AT1R and MasR were incubated with the sections overnight at 4°C, followed by post‐primary and Novolink Polymer incubation. A diaminobenzidine was used to detect the positive immunoreaction and the sections were then counterstained with hematoxylin. A negative control was performed by omitting the primary antibody. The ScanScopeTM CS (Aperio Technologies, CA) was used to obtain the digital images and semiquantitative score.

### 2.8 Western blot

Proteins were extracted from the quadriceps muscle samples using a homogenizing buffer that contained protease and phosphatase inhibitors (*n* = 5). The homogenates were centrifuged at 9700 RPM for 20 min at 4°C, and the supernatant was collected.

The total protein concentration was determined using a BCA protein assay kit (23227, Thermo Scientific, IL, USA). After denaturation at 100°C for 5 min, 50 μg of proteins were loaded onto a polyacrylamide gel (8%–12% SDS‐PAGE) for electrophoresis separation, and the proteins were transferred onto a polyvinylidene difluoride membrane (PVDF, 88518, Thermo Scientific, IL, USA). The blot membranes were blocked with 5% bovine serum albumin for 1 h and then incubated overnight at 4°C with the primary antibodies (1:500, Table S2). The primary antibodies' binding was detected with secondary antibodies (1:10,000, Table S2). Enhanced chemiluminescence reagents (Clarity Western ECL Substrate, 1705061, BioRad, CA, USA) were used to visualize images of the blots in a ChemiDoc MP‐System (BioRad, CA, USA). Finally, the intensity of the bands was quantified using the ImageJ software, version 1.44 (NIH; imagej.nih.gov/ij). Cyclophilin was used as a loading control.

### 2.9 Statistical analysis

Data are presented as means ± standard deviation (SD). The data were tested for normality and homoscedasticity of the variances. The differences among the groups were assessed by analysis of variance (one‐way ANOVA), followed by the Holm–Sidak post hoc test. In all cases, *p* < 0.05 was considered statistically significant. The GraphPad Prism software (version 8.02, La Jolla, CA, USA) was used to perform the statistical analysis.

## 3 RESULTS

### 3.1 Body composition and exercise capacity

The food intake (g), total energy intake (kJ), and body mass (g) evolution of all groups were previously described (Giori et al., 2021; Sepúlveda‐Fragoso et al., 2022). BMI was higher in the HF group after 16 weeks, with an increase of 30.06% (*p* < 0.01) compared to the SC group. However, HF‐E, HF‐T, and HF‐ET groups showed a reduction in BMI when compared to the HF group, with a decrease of 26.88% (*p* < 0.001); 15.19% (*p* < 0.05); and 42.10% (*p* < 0.0001), respectively. Additionally, the HF‐ET group had an even lower BMI compared to the SC (−5.00%, *p* < 0.05) and the HF‐T (−13.79%, *p* < 0.001) groups (Table 1).

**TABLE 1:** Body composition and exercise capacity.

Data	SC	HF	HF‐E	HF‐T	HF‐ET
Body mass index (BMI, g/cm^2^)	0.28 ± 0.05	0.36 ± 0.06**	0.27 ± 0.03^####^	0.31 ± 0.02^#^	0.22 ± 0.03^*####&&&^
Lean mass (DEXA, %)	59.31 ± 8.6	38.13 ± 6.54*	54.94 ± 10.36^#^	49.18 ± 12.02	58.62 ± 4.05^#^
Lean/fat mass (DEXA)	1.68 ± 0.48	0.66 ± 0.19**	1.45 ± 0.52	1.17 ± 0.49	1.56 ± 0.28^#^
Quadriceps muscle mass (mg)	0.33 ± 0.07	0.31 ± 0.04	0.38 ± 0.05	0.41 ± 0.07^##^	0.35 ± 0.05
Triceps surae muscle mass (mg)	0.28 ± 0.03	0.27 ± 0.03	0.28 ± 0.04	0.32 ± 0.04^#^	0.31 ± 0.03
Exhaustion time (min) Week 8	7.16 ± 0.46	8.02 ± 0.52	7.99 ± 0.75	8.02 ± 0.29	7.48 ± 0.33
Week 16	8.24 ± 0.40	7.35 ± 0.41	7.58 ± 0.37	14.67 ± 0.33^a***####†††^	11.00 ± 0.53^a*###††&^
Delta	1.083 ± 0.57	−0.67 ± 0.63	−0.44 ± 0.66	6.65 ± 0.99^**###†††^	3.52 ± 0.45^###††&^

*Note*: Data are presented as mean ± SD, *n* = 10. Significant differences between the groups are indicated by symbols (*p* < 0.05): * ≠ SC; # ≠ HF; † ≠ HF‐E, and & ≠ HF‐T, a ≠ 8th week.

Abbreviations: BMI, body mass index; DEXA, dual‐energy X‐ray absorptiometry; HF, high fat; HF‐E, high‐fat diet treated with enalapril; HF‐ET, high‐fat diet treated with enalapril and exercise training; HF‐T, high‐fat diet, and exercise training; SC, standard chow; SD, standard deviation.

The results of the DEXA analysis showed that the HF group had a lower percentage of lean mass and a lower ratio of lean/fat mass compared to the SC group (−35.71%, *p* = 0.0108; −60.61%, *p* = 0.0099, respectively). However, the HF‐E and HF‐ET groups were able to counteract the negative effects of the HF diet and showed a higher percentage of lean mass compared to the HF group (+44.08%, *p* = 0.05; +53.73%, *p* = 0.0130). In addition, the quadriceps femoris and triceps surae muscle mass were greater in the HF‐T group compared to the HF group (+32.25%, *p* = 0.039; +19.00%, *p* = 0.05, respectively; Table 1).

The time to exhaustion at baseline (data not shown) and after 8 weeks were not significantly different among the groups (Table 1). However, at the end of the study, the trained groups (HF‐T and HF‐ET) had a longer time to exhaustion compared to the sedentary groups (SC, HF, and HF‐E) and their baseline (*p* < 0.05). Additionally, HF‐T was found to be more effective than HF‐ET in improving time to exhaustion (Table 1).

### 3.2 Muscle tissue stereology

**FIGURE 1:**
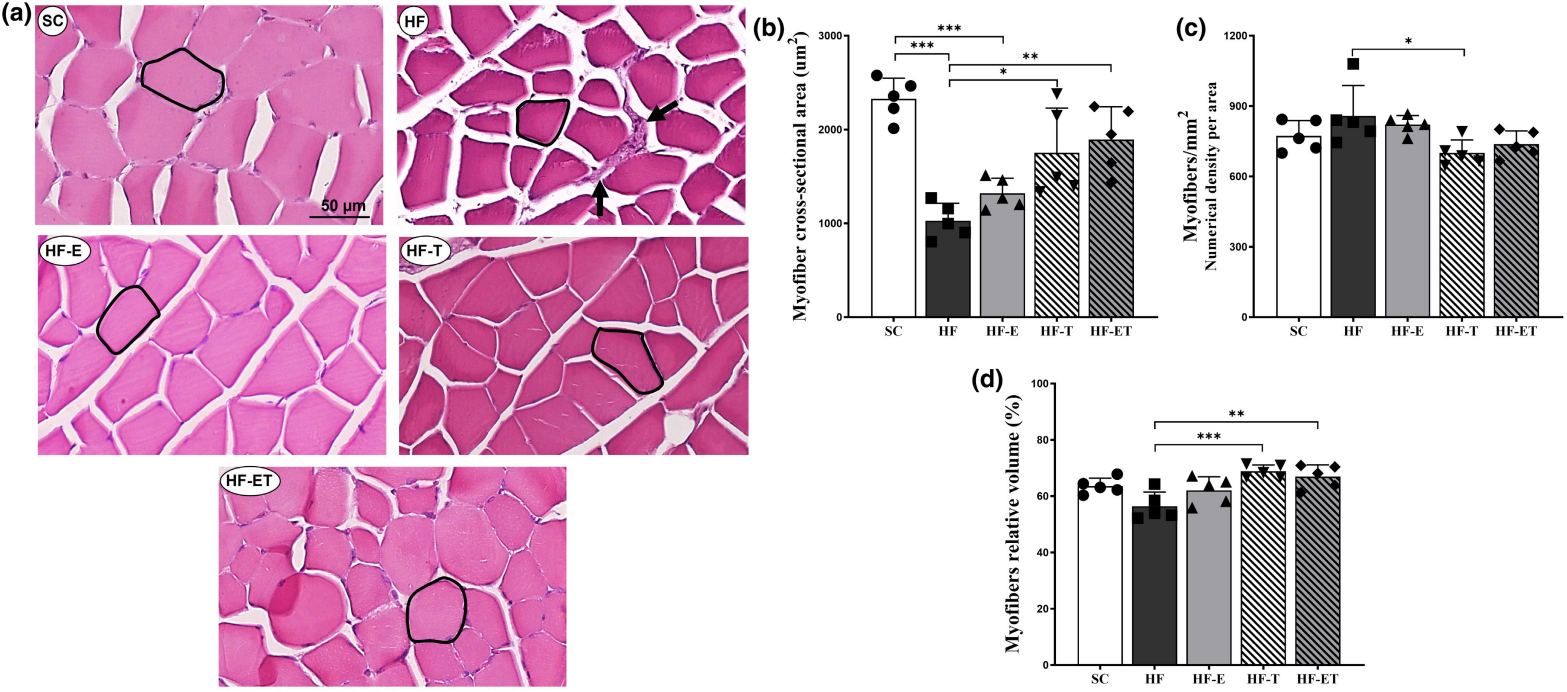
Muscle tissue stereology. (a) Representative images of the myofibers of the experimental groups (bar = 50 μm), stained with hematoxylin–eosin. A myofiber is outlined in each group. SC group showing a normal pattern of skeletal muscle tissue. HF group showing small myofibers, and large interstitial space with cell infiltration (→). (b) Myofiber cross‐sectional area (μm^2^) of the quadriceps muscle. (c) Numerical density of the myofiber per area (1/mm^2^). (d) Volume density of myofibers in percentage. Data are presented as mean ± SD, *n* = 5. Significant differences between groups are indicated in the graphs: *p ≤ 0.05; **p ≤ 0.01; ***p ≤ 0.001, as determined by one‐way ANOVA and Holm–Sidak post‐test.

Histological examination of skeletal muscle was conducted using digital images of a cross‐section of myofibers (Figure 1a). The results of the stereological analysis showed that both the HF and HF‐E groups had impaired myofiber cross‐sectional area (−55.90%, *p* < 0.0001; −43.31%, *p* = 0.0004, respectively) compared to the SC group (Figure 1b). This study found that aerobic exercise training, with or without enalapril, helped reduce myofiber atrophy caused by HF diet consumption by −65.94% (*p =* 0.0119) and − 78.50% (*p =* 0.0021), respectively. The numerical density per area (Qa) showed that the HF group tended to have a higher number of profiles per area compared to the SC group (*p =* 0.075). However, the group that underwent aerobic exercise training (HF‐T) had a lesser number of myofibers per area in the histological sections as compared to the HF group by −18.16% (*p =* 0.036; Figure 1c). Concerning the relative volume occupied by the myofiber in the same area (Vv; Figure 1d), the group that underwent aerobic exercise training, with or without enalapril (HF‐T and HF‐ET), showed an increase in Vv as compared to the HF group (+27.97%, *p* = 0.0008; +17.43%, *p* = 0.0046, respectively). The findings indicate that the HF group exhibited a disarrangement in the muscle histoarchitecture with a reduced less contractile area and increased noncontractile area, comprising large interstitial space. Only the trained groups (HF‐T and HF‐ET) demonstrated an enhancement in muscle repair and contractile area, resembling the morphology of the SC group.

### 3.3 RAS and KKS protein expression

**FIGURE 2:**
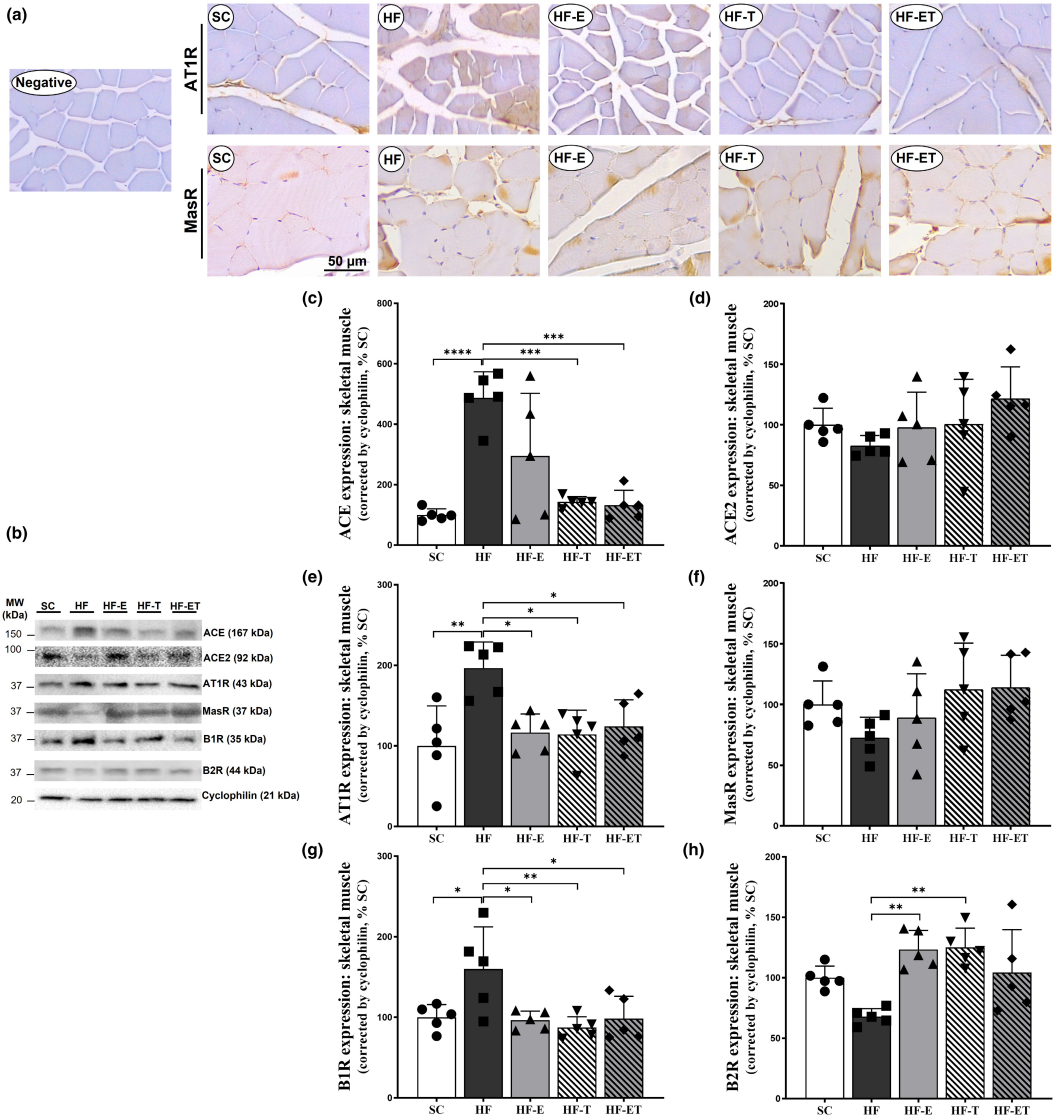
RAS arms. (a) Representative photomicrographs showing myofibers with immunoperoxidase (brown)‐stained AT1R (first line) and MasR (second line), counterstained with hematoxylin, and same magnification in all groups, bar = 50 μm. (b) Representative Western blot analysis with numbers on the left indicates molecular weight (MW, kDa). Protein expression of (c) ACE, (d) ACE2, (e) AT1R, (f) MasR, (g) B1R, (h) B2R in skeletal muscle. Data are presented as mean ± SD, *n* = 5. Significant differences between groups are indicated in the graphs: *p ≤ 0.05; **p ≤ 0.01; ***p ≤ 0.001, ****p ≤ 0.0001, as determined by one‐way ANOVA and Holm–Sidak post‐test.

The RAS classical arm in the skeletal muscle was analyzed. The group that followed a HF diet showed a significant increase in ACE and AT1R protein expressions compared to the SC group (Figure 2b,c,e). Specifically, the increase was +387.19% for ACE (*p* < 0.0001) and + 96.51% for AT1R (*p* = 0.0028). This was confirmed by the immunostaining analysis of muscle tissue (Figure 2a). The HF group showed strong expression for AT1R, while the SC group showed weak expression for MasR. However, the interventions (HF‐E, HF‐T, and HF‐ET) successfully reduced the AT1R protein expression compared to the HF group. The reductions were −40.70% for HF‐E (*p* = 0.0163), −41.83% for HF‐T (*p* = 0.0129), and −36.77% for HF‐ET (*p* = 0.0363) (Figure 2e). Interestingly, only the trained groups (HF‐T and HF‐ET) showed a lower ACE expression than the HF group. The reduction was −70.65% for HF‐T (*p* = 0.0004) and −72.93% for HF‐ET (*p* = 0.0003) (Figure 2c). No significant differences were observed among the experimental groups in ACE2 and MasR in the counterregulatory arm, as shown in Figure 2b,d,f.

Regarding KKS (Figure 2b,g,h), a higher B1R expression was observed in the HF group compared to the SC group (+59.86%, *p* = 0.035). However, all interventions (HF‐E, HF‐T, and HF‐ET) showed a lower B1R expression (−39.75%, *p* = 0.021, −45.38%, *p* = 0.0066; −38.45%, *p* = 0.0273, respectively) than the HF group. Additionally, the HF‐E and HF‐T groups showed a greater expression of B2R (+81.91%, *p* = 0.0021; +84.52%, *p* = 0.0015, respectively) compared to the HF group.

### 3.4 Protein synthesis and mitochondrial biogenesis markers

**FIGURE 3:**
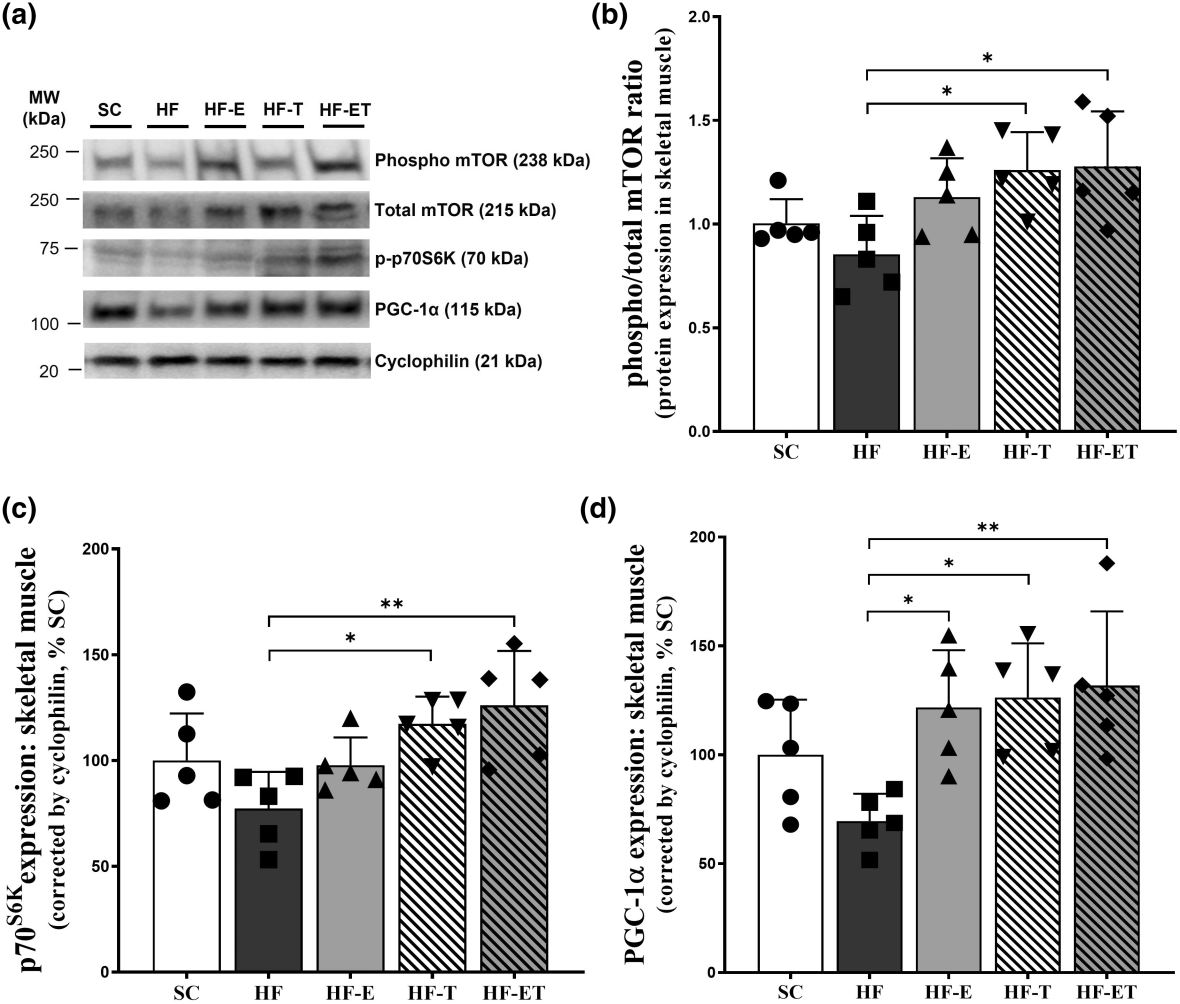
Protein synthesis and mitochondrial biogenesis markers. (a) Representative Western blot analysis with numbers on the left indicates molecular weight (MW, kDa). Protein expression of (b) phosphorylated/total mTOR ratio, (c) phosphorylated p70S6K, and (d) PGC‐1α in skeletal muscle. Data are presented as mean ± SD, *n* = 5. Significant differences between groups are indicated in the graphs: *p ≤ 0.05; **p ≤ 0.01, as determined by one‐way ANOVA and Holm–Sidak post‐test.

The level of protein for phospho‐mTOR/mTOR ratio and p70S6K were similar between the groups that were sedentary (SC), HF, and high‐fat with enalapril (HF‐E), as shown in Figure 3a. However, the trained groups (HF‐T and HF‐ET) showed a higher expression of p‐mTOR/mTOR ratio (+47.54%, *p* = 0.033; +49.65%, *p* = 0.023, respectively; Figure 3b) and p70S6K expression (+51.9%, *p* = 0.0315; +63.26%, *p* = 0.0058, respectively; Figure 3c) compared to the HF group, indicating a greater protein biosynthesis profile.

The expression of PGC‐1α protein did not show any statistical differences between SC and HF groups. However, all the interventions (HF‐E, HF‐T, and HF‐ET) showed higher PGC‐1α expression compared to the HF group (+74.90%, *p* = 0.041; +81.54, *p* = 0.021; +89.39%, *p* = 0.009, respectively; Figure 3d).

### 3.5 Protein degradation and apoptosis markers

**FIGURE 4:**
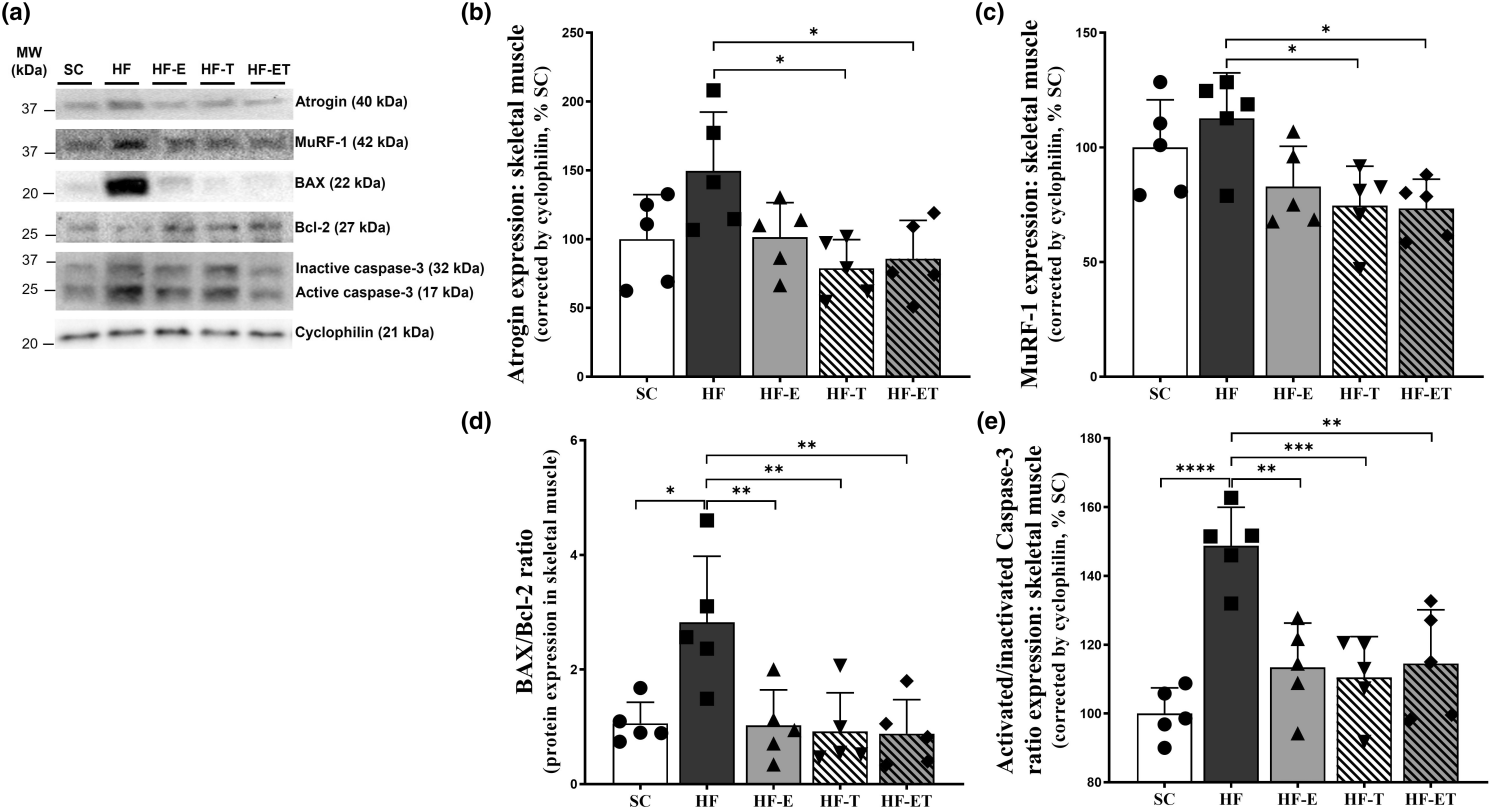
Protein degradation and apoptosis markers. (a) Representative Western blot analysis with numbers on the left indicates molecular weight (MW, kDa). (b) Protein expression of Atrogin‐1, (c) MuRF‐1, (d) BAX/Bcl‐2 ratio, and (e) active/inactive Caspase‐3 in skeletal muscle. Data are presented as mean ± SD, *n* = 5. Significant differences between groups are indicated in the graphs: *p ≤ 0.05; **p ≤ 0.01; ***p ≤ 0.001, ****p ≤ 0.0001, as determined by one‐way ANOVA and Holm–Sidak post‐test.

The present study examined the effects of different interventions on protein degradation markers in skeletal muscle (Figure 4a). We showed that the HF‐T group had lower Atrogin‐1 (−47.31%, *p* = 0.016) and MuRF‐1 (−33.80%, *p* = 0.0297) protein expressions, as well as the HF‐ET group (Atrogin‐1: −42.67%, *p* = 0.0361, Figure 4b; MuRF‐1: −34.90%, *p* = 0.0232, Figure 4c), both compared to the HF group.

Additionally, the HF group had a higher BAX/Bcl‐2 ratio (Figure 4d) and active/inactive caspase‐3 expression ratio (Figure 4e) than the SC group (+166.41%, *p* = 0.0104; +48.76%, *p* < 0.0001, respectively), indicating the activation of the pro‐apoptotic pathway. However, all interventions (HF‐E, HF‐T, and HF‐ET) showed a lower BAX/Bcl‐2 ratio (−63.69%, *p* = 0.0089; −67.48%, *p* = 0.0051; −68.80%, *p* = 0.0042, respectively) and active/inactive caspase‐3 ratio (−23.76%, *p* = 0.0016; −25.71%, *p* = 0.0007; −23.02%, *p* = 0.0023, respectively) compared to the HF group. This suggests that these interventions favor the anti‐apoptotic pathway.

## 4 DISCUSSION

In this study, we conducted a comparison of the effects of enalapril and aerobic exercise training, both individually and in combination, on the skeletal muscle remodeling caused by obesity. Our study yielded three main findings: (1) the use of enalapril alone led to an improvement in body composition, decreased the levels of apoptosis markers, and increased mitochondrial biogenesis markers in skeletal muscle; (2) exercise training was more effective than enalapril in reducing myofiber atrophy caused by a HF diet; (3) enalapril did not contribute to any enhancement in training outcomes, signaling, or muscle morphology.

Obesity can impair skeletal muscle mass and function, due to an imbalance in the process of protein synthesis and degradation. Previous studies have produced conflicting results regarding the relationship between obesity and mTOR signaling (França et al., 2020; Han et al., 2023; Woo et al., 2016). Nilsson et al. showed that obese animals had a blunted protein synthesis response to exercise, indicating that their muscle had become desensitized to the anabolic stimulus of exercise (Nilsson et al., 2013). In addition, the muscle of individuals with obesity seems to be resistant to the anabolic action of targeted exercise regimes when compared to normal‐weight adults. This suggests that muscle protein remodeling in response to the main anabolic stimuli to human skeletal muscle tissue is impaired in obesity (Beals et al., 2019). Obesity not only impairs protein synthesis rates but also leads to skeletal muscle atrophy through several mechanisms, such as oxidative stress, apoptosis, mitochondrial dysfunction, and UPP overactivation (Abrigo et al., 2016; Heo et al., 2018).

The overactivation of the classical arm of the RAS is one of the molecular mechanisms that cause muscle dysfunction in obese individuals (Frantz et al., 2018). Ang II, a molecule that promotes protein catabolism, activates several proteins, including caspase‐3 and the UPP complex, which leads to muscle loss. However, this effect can be reduced by activating the counterregulatory arm (Brink et al., 2001; Meneses et al., 2015). Our study has shown that using enalapril and aerobic exercise training, either alone or in combination, can effectively reduce the expression of components of the classical RAS axis. These treatments also can shift KKS balance toward the B2R in skeletal muscle. The ACE2/Ang (1–7)/MasR arm plays a critical role in preventing muscle wasting (Frantz et al., 2018; Giori et al., 2021), like how ACE2 deficiency may impair physical performance and skeletal muscle adaptations to exercise (Motta‐Santos et al., 2016). The expression of B2R over B1R also protects against diet‐induced obesity, muscle inflammation, glucose tolerance, and aerobic exercise performance (Morais et al., 2015; Reis et al., 2015). ACEi, like enalapril, may help preserve high‐affinity B2R and block B2R desensitization and internalization by potentializing bradykinin (Minshall et al., 1997; Su, 2014). Previous studies have investigated muscle adaptation to exercise in obesity (Woo et al., 2016; de Sousa et al., 2021; Frantz et al., 2017), and we demonstrated that exercise training prevents the activation of the B1R pathway and preserves B2R.

Enalapril has been found to have a positive effect on glucose metabolism and the reduction of body adiposity. This is believed to be due its ability to increase fat oxidation by raising adiponectin levels, improving peroxisome proliferator‐activated receptor (PPAR)‐γ signaling, and enhancing muscle mitochondrial function (Carter et al., 2011; Frantz et al., 2013; Giori et al., 2021; Santos et al., 2008). In this study, we have demonstrated that enalapril helps in increasing the expression of PGC‐1α, while reducing the markers of muscle apoptosis (such as BAX/Bcl‐2 ratio and caspase‐3). However, it has no effect on the markers of protein biosynthesis (such as mTOR and p‐p70S6K) or UPP (such as Atrogin‐1 and MuRF‐1) in skeletal muscle. Enalapril also reduces mitochondrial DNA oxidation, which explains the higher content of PGC‐1α and TFAM in cardiac muscle. This suggests an upregulation of mitochondrial biogenesis under ACE inhibition (Picca et al., 2018). Many studies have reported that enalapril protects against muscle deterioration by reducing oxidative stress, cell death, and the BAX/Bcl‐2 ratio. It also reduces caspase‐dependent apoptotic pathway signaling (Carter et al., 2011; Marzetti et al., 2013). However, it is difficult to determine whether the improvement in cardiovascular health resulting from ACEi, is due to its impact on adiposity (Alexandre‐Santos et al., 2022; Giori et al., 2021) or it is a direct effect on HF diet‐induced obesity.

Aerobic exercise training is a helpful tool for improving the quality of life and reducing the risk of morbidity (Hawley et al., 2021). Endurance exercise training leads to various metabolic and morphological changes, such as mitochondrial biogenesis (Coffey & Hawley, 2007). Endurance exercise is known for stimulating PGC‐1α, which is believed to be the master regulator of mitochondrial biogenesis, muscle oxidative capacity, and protein metabolism (Millay & Olson, 2013). Improvements in mitochondrial metabolism may contribute to skeletal muscle contractility and anabolism after aerobic exercise training (Konopka & Harber, 2014). In this study, exercise training improved PGC‐1α, mTOR, and p‐p70S6K muscle protein expression compared to the HF group. These findings are consistent with some studies that have shown an increase in mTOR expression, 24 and 48 h after endurance treadmill exercise, without hypertrophy of muscle fiber (França et al., 2020; Pagano et al., 2014; Suzuki, 2019). Instead, these results may indicate increased protein translation and synthesis during recovery, contributing to enhanced mitochondrial function, exercise capacity and counteracting obesity‐related muscle mass loss in the trained HF group. Furthermore, exercise training not only enhances muscle biosynthesis but also reduces markers of muscle degradation, such as Atrogin‐1 and MuRF‐1, and protects against early mitochondria‐mediated apoptotic signaling caused by obesity (BAX/Bcl‐2 ratio and caspase‐3 expression) (Harber et al., 2012; Konopka & Harber, 2014). Our research findings support these claims, as we observed the downregulation of UPP pathways and apoptotic markers in trained groups, mitigating obesity‐induced myofiber atrophy.

The combination of interventions was effective in reducing adiposity and the ratio of fat/lean mass by BMI and DEXA. However, they did not work together to improve obesity‐induced skeletal muscle remodeling. Enalapril treatment alone did not impact skeletal muscle mass or exercise capacity in untrained obese mice (HF‐E group). However, when enalapril was combined with exercise (HF‐ET group) it led to impaired quadriceps muscle mass and exhaustion time compared to isolated training (HF‐T). These findings suggest that physical performance worsened in the HF‐ET group, even though we did not observe any difference in trained groups regarding molecular pathways. The effects of ACEi on physical performance are contradictory, in various studies, reported as being impaired (Minami et al., 2004; Sjúrðarson et al., 2022), enhanced (Minami et al., 2007), or unchanged (Bahi et al., 2004; Carter et al., 2011). Although the mechanism behind these findings is not clear, the hypothesis supported by Minami et al. (2004, 2007) is that the ACEi activate the bradykinin/nitric oxide (NO) pathway by inhibiting the skeletal muscle mitochondrial respiration and impairing the higher exercise capacity acquired by training. On the other hand, beneficial mechanisms have been proposed, including improved glucose sensitivity, endothelial function, and reduced inflammation (Wang et al., 2008; Bahi et al., 2004; Sjúrðarson et al., 2022). However, no studies have examined the effects of ACEi on exercise capacity acquired by exercise training in the obesity model.

When interpreting the results of this study, it is important to acknowledge some specific limitations. The inclusion of enalapril in the manufactured diet, maintaining an average dose of 10 mg/kg/day, without standardization or adjustment during treatment, poses a constraint to our study. Another key limitation lies in the exclusive focus on the quadriceps muscle for molecular analysis, despite running engaging a diverse group of hindlimb muscles, such as the quadriceps, gastrocnemius, and soleus muscles. Furthermore, the absence of determination regarding muscle fiber type, given the predominance of type II fibers in the quadriceps, represents a limitation. While the study hints at alterations in the expression of the RAS components in skeletal muscle, it is imperative to underscore the importance of conducting functional assessments and downstream mechanism studies to a more comprehensive understanding of pathways involved in obesity‐induced muscle remodeling.

## 5 CONCLUSIONS

Our study indicates that both enalapril and exercise training, whether administered alone or in combination, effectively attenuate the expression of the components of the classical RAS axis. This promotes the expression of B2R over B1R and mitigates apoptosis markers. Notably, exercise training alone emerged as the most effective strategy for counteracting obesity‐induced skeletal muscle remodeling, fostering increased muscle mass, activating biosynthesis pathways, and concurrently reducing skeletal muscle UPP activation.

Interestingly, our findings indicate that the combined approach of enalapril and exercise lacks a synergistic effect in combating obesity‐induced skeletal muscle remodeling, and it may even compromise the acquired exercise capacity. This underscores the need for further investigations to unravel the intricate mechanisms governing the interaction between aerobic exercise training and ACEi in the context of the obesity model.

## AUTHOR CONTRIBUTIONS

ABP, IGG, ACLN, and EDCF were involved in the conception and design of the research; ABP, JAMSF, IGG, BAS, CMS, DCM, and EDCF collected data, performed the statistical analysis, and drafted the paper; ABP, JAMSF, BAS, CMS, DCM, MM, ACLN, and EDCF edited and revised the manuscript. All authors read and approved the final manuscript.

## ACKNOWLEDGMENTS

The authors thank the laboratory team, and Karin Soares Gonçalves Cunha, Nathalia Silva Oliveira, Bernardo Acácio and Wagner de Souza Rodrigues for their assistance.

## FUNDING INFORMATION

This work was supported by the agencies FAPERJ (Rio de Janeiro State Foundation for Scientific Research, www.faperj.br) [grant numbers E‐26/010.001841/2019 and E‐26/201.347/2022 to EDCF, E‐26/010.002170/2019 to ACLN, and E26/211.280/2019 to DCM]; CNPq (Brazilian Council of Science and Technology, www.cnpq.br) [grant number 421753/2018‐8 to DCM]; and CAPES [Coordenação de Aperfeicoamento de Pessoal de Nivel Superior—Brazil—Finance Code 001]. This research has received partial funding from the Ross University School of Veterinary Medicine.

## CONFLICT OF INTEREST STATEMENT

The authors declare no conflict of interest.

## ETHICS STATEMENT

All the experimental procedures were conducted following conventional guidelines for animal experimentation (National Institute of Health Guide, 8th edition, 2011) and approved by the Animal Ethics Committee of the Fluminense Federal University (Protocol number CEUA 2504060718). Guidelines Checklist: ARRIVE.

## Supporting information


Table S1.

Table S2.

## Data Availability

The corresponding author will provide the data upon reasonable request, subject to privacy and ethical restrictions.
