# Supplementary material for: Obesity‐induced skeletal muscle remodeling: A comparative analysis of exercise training and ACE‐inhibitory drug in male mice
Source: Physiol Rep. 2024 Apr 29;12(9):e16025. doi: 10.14814/phy2.16025 (PMC11058004; doi:10.14814/phy2.16025)
Supplement: Supplementary file 1 — Table S1. [file PHY2-12-e16025-s002.docx]

**Supplementary Table S1:** Detailed composition of the diets.

| **Nutrients** | **Diets (g/kg)** | |
| --- | --- | --- |
|  | **SC** | **HF** |
| Casein | 140.0 | 175.0 |
| Corn starch | 466.0 | 193.0 |
| Dextrinized starch | 155.0 | 155.0 |
| Sucrose | 100.0 | 100.0 |
| Soybean oil | 40.0 | 40.0 |
| Lard | - | 238.0 |
| Fiber | 50.0 | 50.0 |
| Vitamin mix^*^ | 10.0 | 10.0 |
| Mineral mix^*^ | 35.0 | 35.0 |
| Cystine | 1.8 | 1.8 |
| Choline | 2.5 | 2.5 |
| Antioxidant | 0.008 | 0.060 |
| Energy (kJ/g) | 15.0 | 21.0 |
| Carbohydrates (%, energy) | 76 | 36 |
| Protein (%, energy) | 14 | 14 |
| Lipids (%, energy) | 10 | 50 |

*Mineral and vitamin mixtures are following AIN93M.
