# Supplementary material for: Obesity‐induced skeletal muscle remodeling: A comparative analysis of exercise training and ACE‐inhibitory drug in male mice
Source: Physiol Rep. 2024 Apr 29;12(9):e16025. doi: 10.14814/phy2.16025 (PMC11058004; doi:10.14814/phy2.16025)
Supplement: Supplementary file 2 — Table S2. [file PHY2-12-e16025-s001.docx]

**Supplementary Table S2:** Primary and secondary antibodies used in Western blot assay.

| **Antibody** | **Company** | **Code** |
| --- | --- | --- |
| ACE | Abcam | ab11734 |
| ACE2 | Abcam | ab108252 |
| AT1R | Santa Cruz Biotechnology | sc-579 |
| MasR | Santa Cruz Biotchenology | sc-390453 |
| B1R | Santa Cruz Biotechnology | sc-25484 |
| B2R | Santa Cruz Biotechnology | sc-25671 |
| p-P70S6k | Santa Cruz Biotechnology | sc-11759 |
| Atrogin-1 | Cloud-Cone | PAF435Hu01 |
| MuRF-1 | FineTest | FNab08992 |
| Caspase-3 | Cusabio | csb-PA001231 |
| mTOR | Santa Cruz Biotechnology | sc-8319 |
| p-mTOR | Santa Cruz Biotechnology | sc-293132 |
| PGC-1α | Santa Cruz Biotechonoly | sc-518025 |
| BAX | Novus Biologicals | NBP1-28566 |
| Bcl-2 | Cusabio | csb-MA000196 |
| Cyclophilin | Santa Cruz Biotechnology | sc-20361 |
| Anti-rabbit | Santa Cruz Biotechnology | sc-2357 |
| Anti-goat | Santa Cruz Biotechnology | sc-2354 |
| Anti-mouse | Santa Cruz Biotechnology | sc-516102 |
